# Supplementary material for: Isolation of lactic acid bacteria capable of reducing environmental alkyl and fatty acid hydroperoxides, and the effect of their oral administration on oxidative-stressed nematodes and rats
Source: PLoS One. 2020 Feb 27;15(2):e0215113. doi: 10.1371/journal.pone.0215113 (PMC7046221; doi:10.1371/journal.pone.0215113)
Supplement: S1 Fig — The distribution of eliminating activities for hydrogen peroxide in bacterial strains including lactic acid bacteria and the difference in their capacities. Twenty lactic acid bacterial strains and typical bacterial species were cultured under specific conditions described in the Materials and Methods section. Each living cell was exposed to various concentrations of hydrogen peroxide at 0.3, 1.0, and 3.0 mM for 1.5 h. After treatment, the extent of decomposition of hydrogen peroxide, as their eliminating activity, was determined by calculating the difference between the initial concentration and the remaining concentration in the culture medium. The bar graph represents the mean values from three independent experiments, and error bars indicate the standard deviation (SD). (PPTX) [file pone.0215113.s001.pptx]

## Slide 1
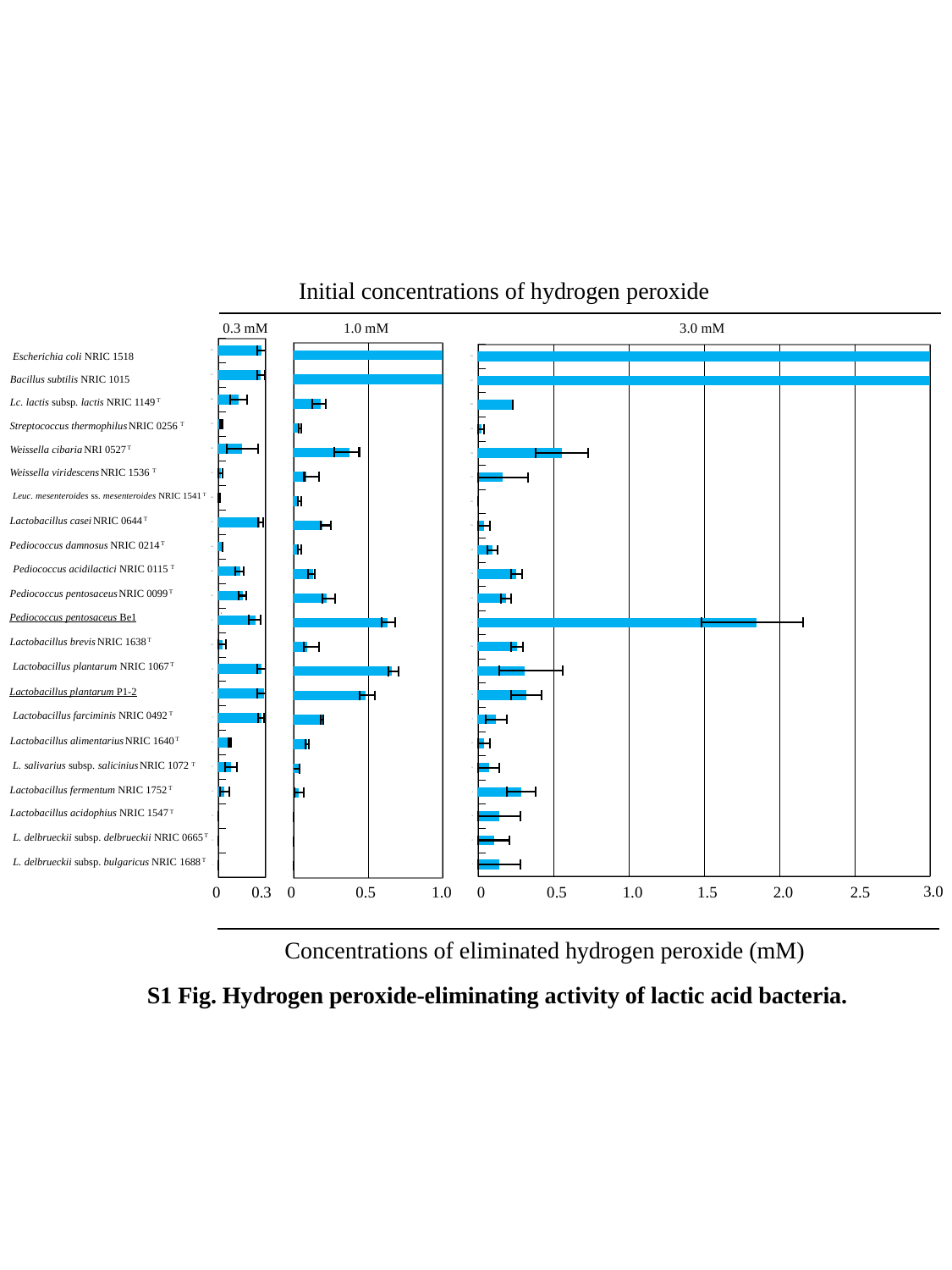

Initial concentrations of hydrogen peroxide
0.3 mM
3.0 mM
1.0 mM
### Chart
| Category | |
|---|---|
### Chart
| Category | |
|---|---|
### Chart
| Category | |
|---|---|
### Chart
| Category | |
|---|---|Escherichia coli NRIC 1518
Bacillus subtilis NRIC 1015
Lc. lactis subsp. lactis NRIC 1149 T
Streptococcus thermophilus NRIC 0256 T
Weissella cibaria NRI 0527 T
Weissella viridescens NRIC 1536 T
Leuc. mesenteroides ss. mesenteroides NRIC 1541 T
Lactobacillus casei NRIC 0644 T
Pediococcus damnosus NRIC 0214 T
Pediococcus acidilactici NRIC 0115 T
Pediococcus pentosaceus NRIC 0099 T
Pediococcus pentosaceus Be1
Lactobacillus brevis NRIC 1638 T
Lactobacillus plantarum NRIC 1067 T
Lactobacillus plantarum P1-2
Lactobacillus farciminis NRIC 0492 T
Lactobacillus alimentarius NRIC 1640 T
L. salivarius subsp. salicinius NRIC 1072 T
Lactobacillus fermentum NRIC 1752 T
Lactobacillus acidophius NRIC 1547 T
L. delbrueckii subsp. delbrueckii NRIC 0665 T
L. delbrueckii subsp. bulgaricus NRIC 1688 T
3.0
0
0.3
0
0.5
1.0
0
0.5
1.0
1.5
2.0
2.5
Concentrations of eliminated hydrogen peroxide (mM)
S1 Fig. Hydrogen peroxide-eliminating activity of lactic acid bacteria.
