# Supplementary material for: Isolation of lactic acid bacteria capable of reducing environmental alkyl and fatty acid hydroperoxides, and the effect of their oral administration on oxidative-stressed nematodes and rats
Source: PLoS One. 2020 Feb 27;15(2):e0215113. doi: 10.1371/journal.pone.0215113 (PMC7046221; doi:10.1371/journal.pone.0215113)
Supplement: S2 Fig — Before and after heated cell were exposed 3.0 mM cumene hydroperoxide or 1.0 mM linoleic acid hydroperoxide for 3.0 h. The cell suspension was boiled at 100°C for 10min in the heated treatment. The bar graph represents the mean values from three independent experiments. (PPTX) [file pone.0215113.s002.pptx]

## Slide 1
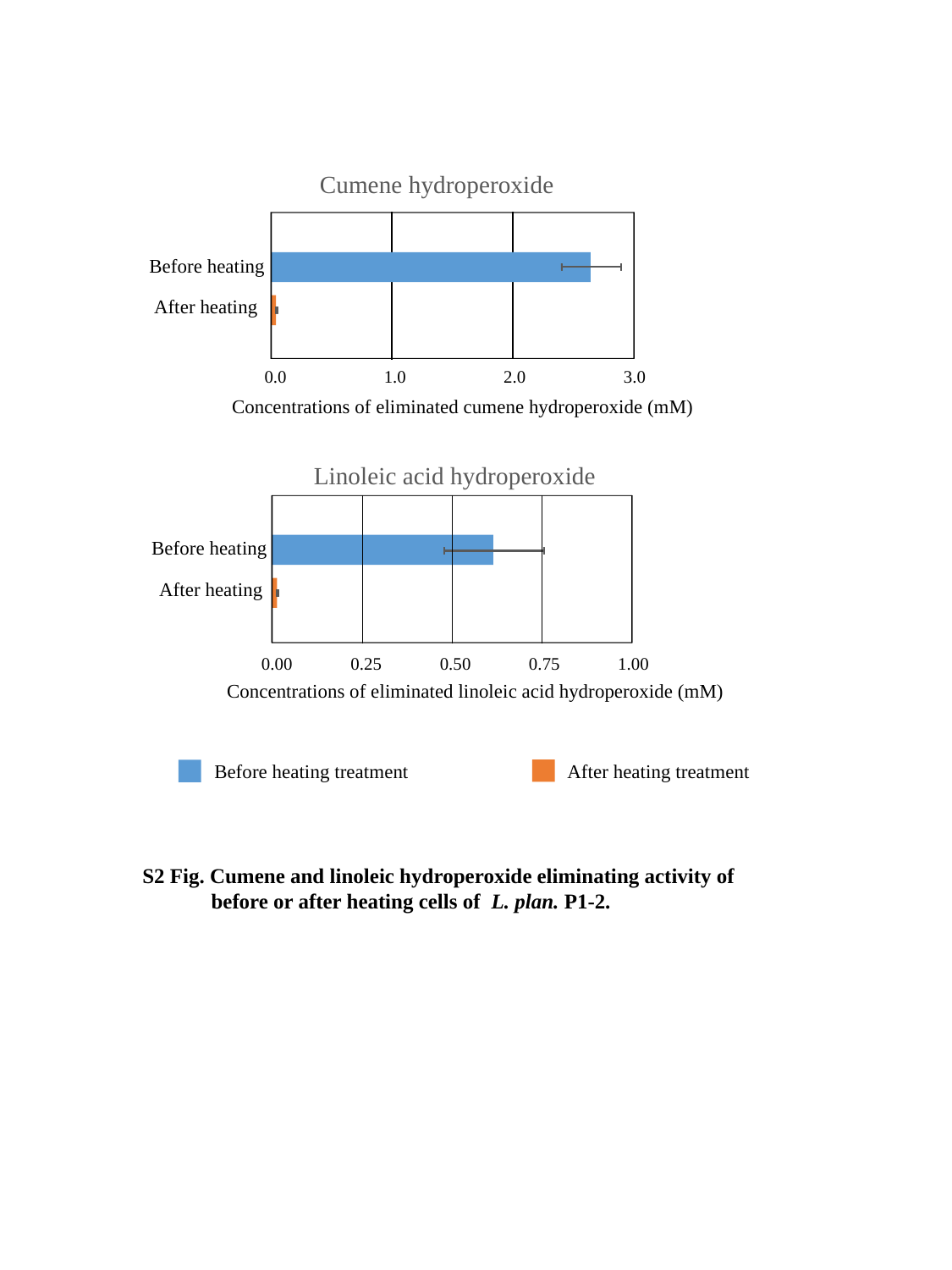

Cumene hydroperoxide
Before heating
After heating
0.0
1.0
2.0
3.0
Concentrations of eliminated cumene hydroperoxide (mM)
Linoleic acid hydroperoxide
Before heating
After heating
0.00
0.25
0.50
0.75
1.00
Concentrations of eliminated linoleic acid hydroperoxide (mM)
Before heating treatment
After heating treatment
S2 Fig. Cumene and linoleic hydroperoxide eliminating activity of
 before or after heating cells of L. plan. P1-2.
