# Supplementary material for: Isolation of lactic acid bacteria capable of reducing environmental alkyl and fatty acid hydroperoxides, and the effect of their oral administration on oxidative-stressed nematodes and rats
Source: PLoS One. 2020 Feb 27;15(2):e0215113. doi: 10.1371/journal.pone.0215113 (PMC7046221; doi:10.1371/journal.pone.0215113)
Supplement: S3 Fig — Cumene hydroperoxide reduction to 2-phenyl-2-propanol by L. plantarum P1-2 was presented as a bar graph. Cumene hydroperoxide and 2-phenyl-2-propanol were analyzed with HPLC as described in the Materials and Methods section. The bar graph represents the mean values from two independent experiments, and error bars indicate the standard deviation (SD). (PPTX) [file pone.0215113.s003.pptx]

## Slide 1
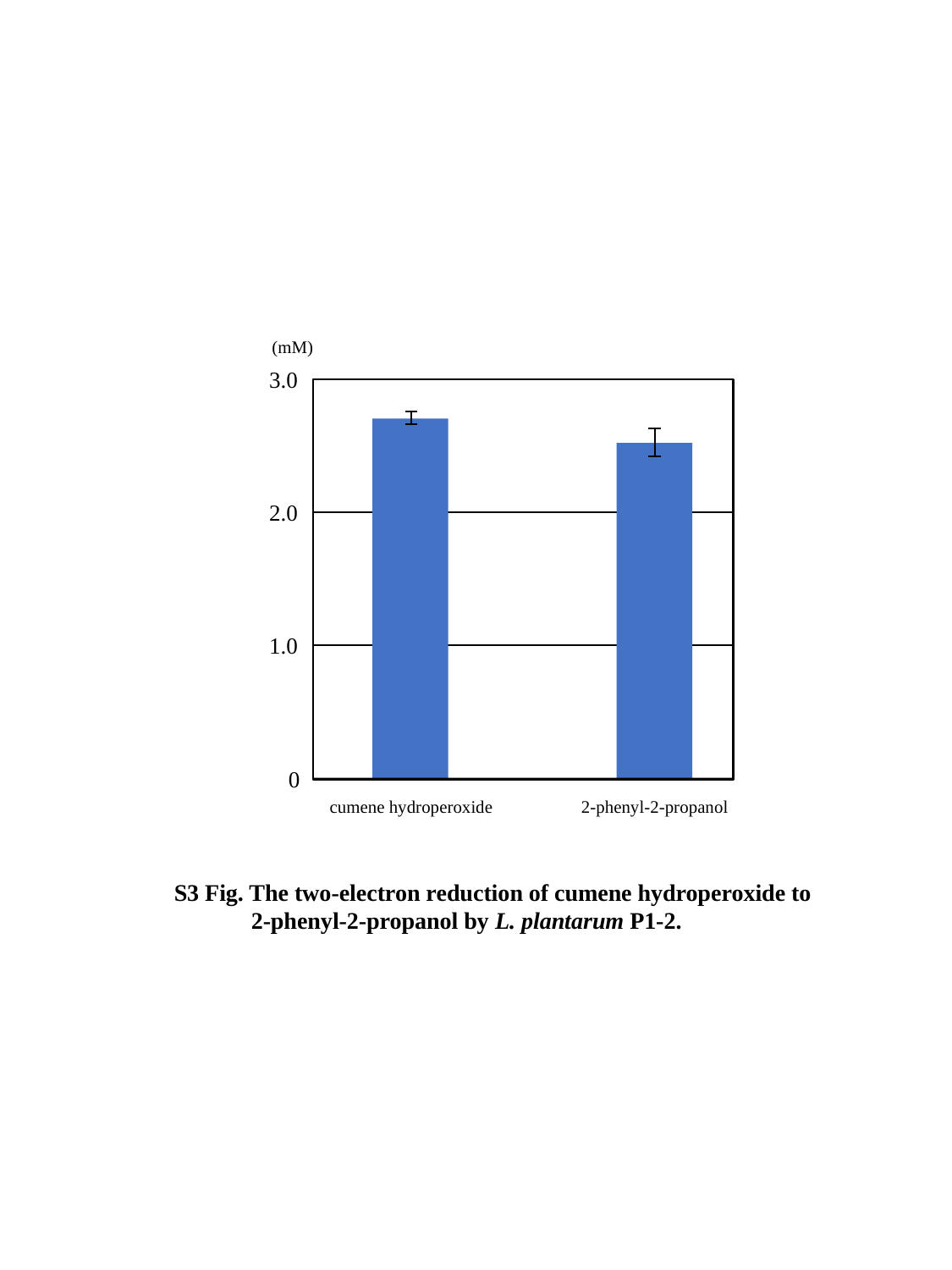

(mM)
3.0
2.0
1.0
0
cumene hydroperoxide
2-phenyl-2-propanol
S3 Fig. The two-electron reduction of cumene hydroperoxide to
 2-phenyl-2-propanol by L. plantarum P1-2.
