# Supplementary material for: Isolation of lactic acid bacteria capable of reducing environmental alkyl and fatty acid hydroperoxides, and the effect of their oral administration on oxidative-stressed nematodes and rats
Source: PLoS One. 2020 Feb 27;15(2):e0215113. doi: 10.1371/journal.pone.0215113 (PMC7046221; doi:10.1371/journal.pone.0215113)
Supplement: S4 Fig — Resistant ability of L. plantarum P1-2 (red), and P. pentosaceus Be1 (blue) against artificial gastric juice and intestinal fluid. The bacterial suspensions before and after 2 h-treatment with artificial gastric juice were incubated on MRS agar. Also, the bacterial suspensions before and after 18 h-treatment with artificial intestinal fluid were incubated on MRS agar. The point represents the mean values from average of two independent experiments. (PPTX) [file pone.0215113.s004.pptx]

## Slide 1
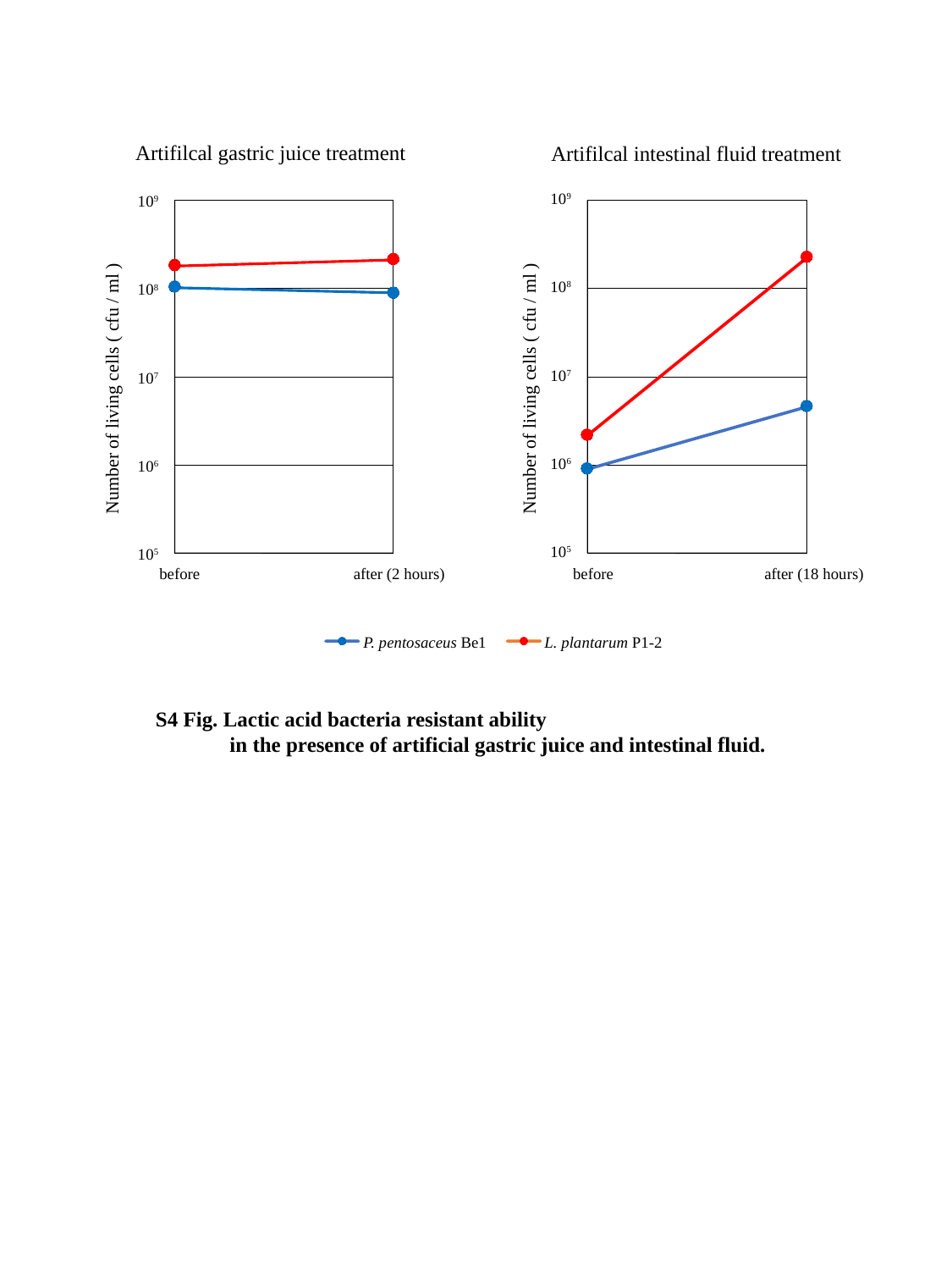

Artifilcal gastric juice treatment
Artifilcal intestinal fluid treatment
109
109
108
108
107
107
Number of living cells ( cfu / ml )
Number of living cells ( cfu / ml )
106
106
105
105
before
after (18 hours)
before
after (2 hours)
P. pentosaceus Be1
L. plantarum P1-2
S4 Fig. Lactic acid bacteria resistant ability
 in the presence of artificial gastric juice and intestinal fluid.
