# Supplementary material for: Isolation of lactic acid bacteria capable of reducing environmental alkyl and fatty acid hydroperoxides, and the effect of their oral administration on oxidative-stressed nematodes and rats
Source: PLoS One. 2020 Feb 27;15(2):e0215113. doi: 10.1371/journal.pone.0215113 (PMC7046221; doi:10.1371/journal.pone.0215113)
Supplement: S5 Fig — E. coli OP50 (gray), S. thermophiles NRIC0256T (green), L. plantarum P1-2 (red), and P. pentosaceus Be1 (blue) were administered to C. elegans Δfer-15 when the growth stage reached L4. The mutants were hatched on pH stat GYP medium, and their lifespan was monitored until annihilation. Statistical analysis was carried out by student’s t-test and Tukey’s multiple-range test. The least significant difference test was used for means separation at P < 0.05 within each strain. One hundred animals were measured for each strain at 25°C. (PPTX) [file pone.0215113.s005.pptx]

## Slide 1
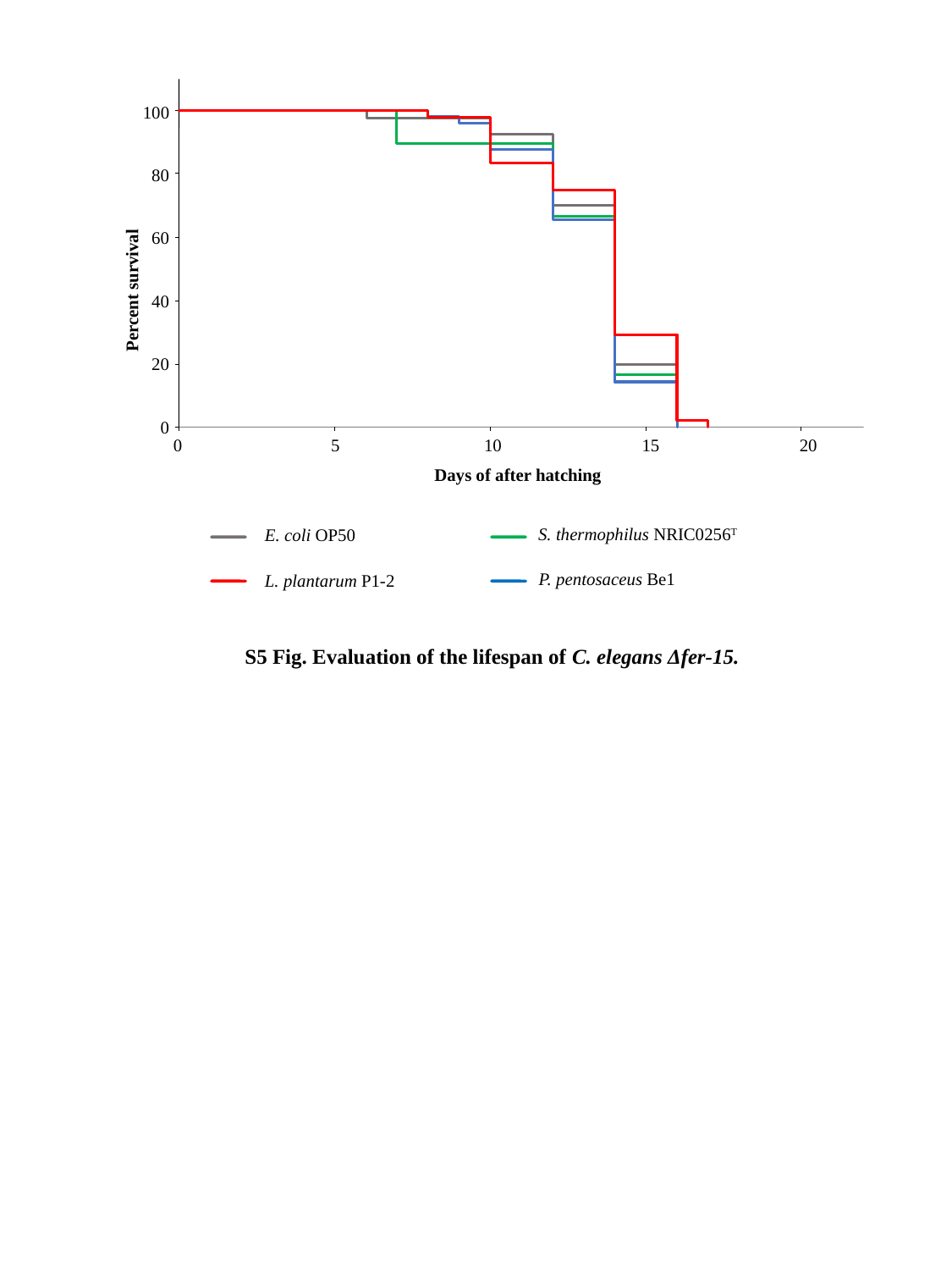

100
80
60
Percent survival
40
20
0
0
5
10
15
20
Days of after hatching
S. thermophilus NRIC0256T
E. coli OP50
P. pentosaceus Be1
L. plantarum P1-2
S5 Fig. Evaluation of the lifespan of C. elegans Δfer-15.
