# Supplementary material for: Isolation of lactic acid bacteria capable of reducing environmental alkyl and fatty acid hydroperoxides, and the effect of their oral administration on oxidative-stressed nematodes and rats
Source: PLoS One. 2020 Feb 27;15(2):e0215113. doi: 10.1371/journal.pone.0215113 (PMC7046221; doi:10.1371/journal.pone.0215113)
Supplement: S6 Fig — P. pentosaceus Be1 was administered to iron-overloaded rats, and the MDA levels in the colonic mucosa were compared to those of the healthy (control) and iron-overloaded rats (Fe). L. plantarum P1-2 and S. thermophilus NRIC0256T were also tested as the control strain. The data are the mean values ± SD (n = 4). (PPTX) [file pone.0215113.s006.pptx]

## Slide 1
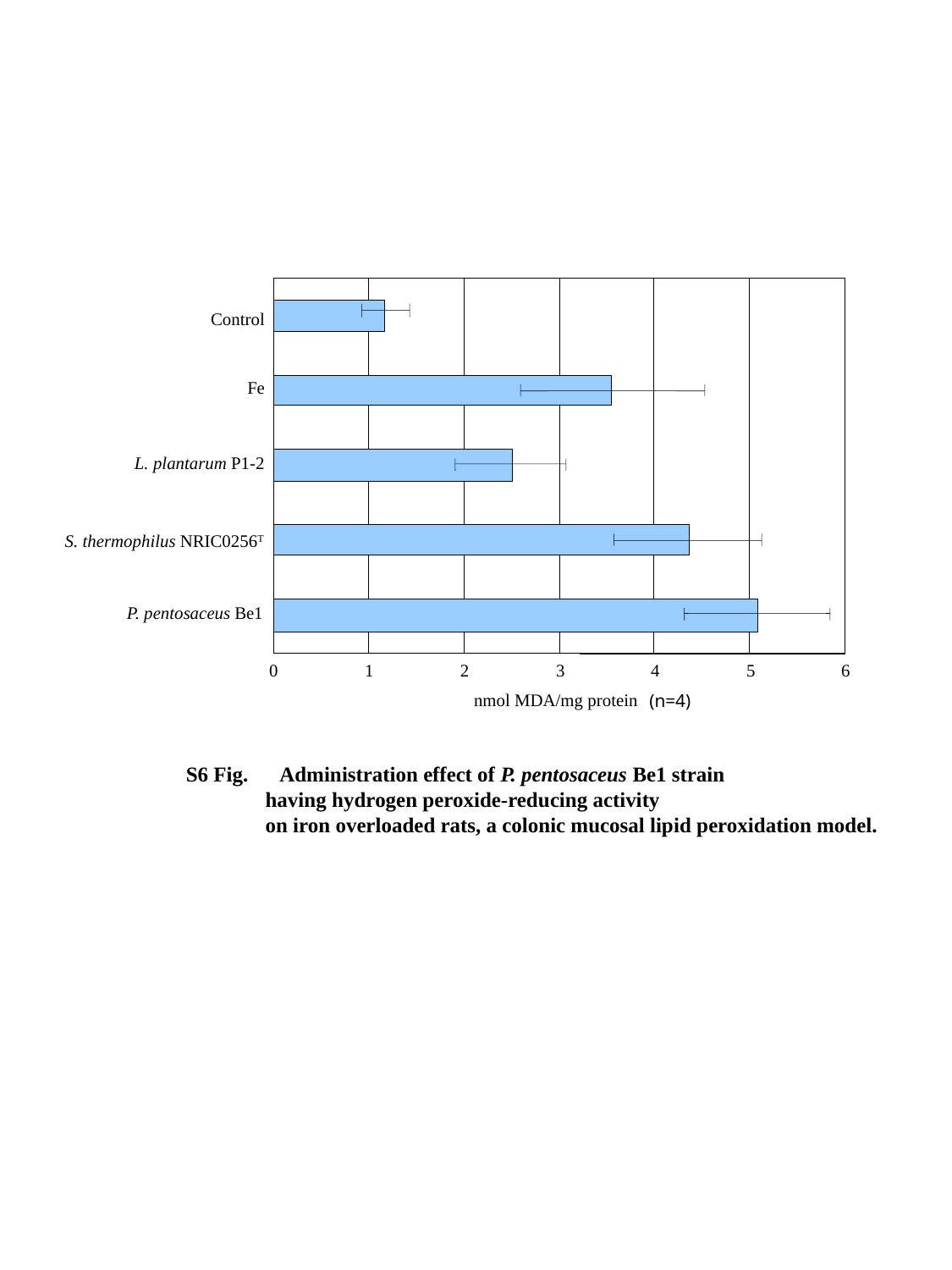

Control
Fe
L. plantarum P1-2
S. thermophilus NRIC0256T
P. pentosaceus Be1
0
1
2
3
4
5
6
(n=4)
nmol MDA/mg protein
S6 Fig.　Administration effect of P. pentosaceus Be1 strain
 having hydrogen peroxide-reducing activity
 on iron overloaded rats, a colonic mucosal lipid peroxidation model.
