# Supplementary material for: Isolation of lactic acid bacteria capable of reducing environmental alkyl and fatty acid hydroperoxides, and the effect of their oral administration on oxidative-stressed nematodes and rats
Source: PLoS One. 2020 Feb 27;15(2):e0215113. doi: 10.1371/journal.pone.0215113 (PMC7046221; doi:10.1371/journal.pone.0215113)
Supplement: S7 Fig — Heat-treated dead L. plantarum P1-2 was administered to iron-overloaded rats, and the MDA levels in the liver and colonic mucosa were compared to those of the healthy (control), iron-overloaded rats (Fe). Heat-treated dead S. thermophilus NRIC0256T was also tested as the control strain. Heat treatment performed lactic acid bacteria culture mediums were boiled at 100°C, 10min for sterilization before centrifugation and lyophilization. The data are the mean values ± SD (n = 3). (PPTX) [file pone.0215113.s007.pptx]

## Slide 1
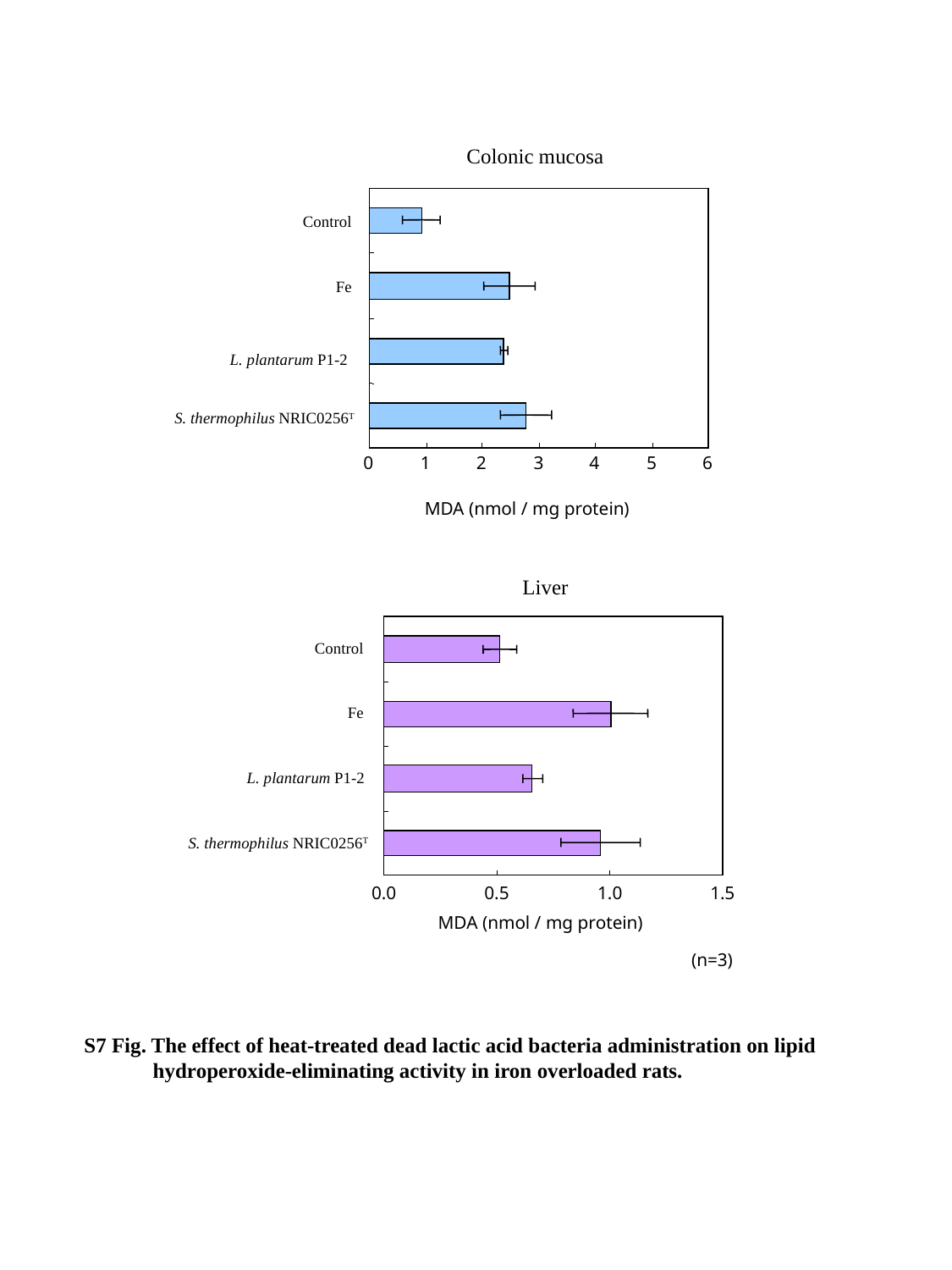

Colonic mucosa
Control
Fe
0
1
2
3
4
5
6
MDA (nmol / mg protein)
L. plantarum P1-2
S. thermophilus NRIC0256T
Liver
Control
Fe
L. plantarum P1-2
S. thermophilus NRIC0256T
0.0
0.5
1.0
1.5
MDA (nmol / mg protein)
(n=3)
S7 Fig. The effect of heat-treated dead lactic acid bacteria administration on lipid
 hydroperoxide-eliminating activity in iron overloaded rats.
