# Supplementary material for: Isolation of lactic acid bacteria capable of reducing environmental alkyl and fatty acid hydroperoxides, and the effect of their oral administration on oxidative-stressed nematodes and rats
Source: PLoS One. 2020 Feb 27;15(2):e0215113. doi: 10.1371/journal.pone.0215113 (PMC7046221; doi:10.1371/journal.pone.0215113)
Supplement: S1 Table — (PPTX) [file pone.0215113.s008.pptx]

## Slide 1
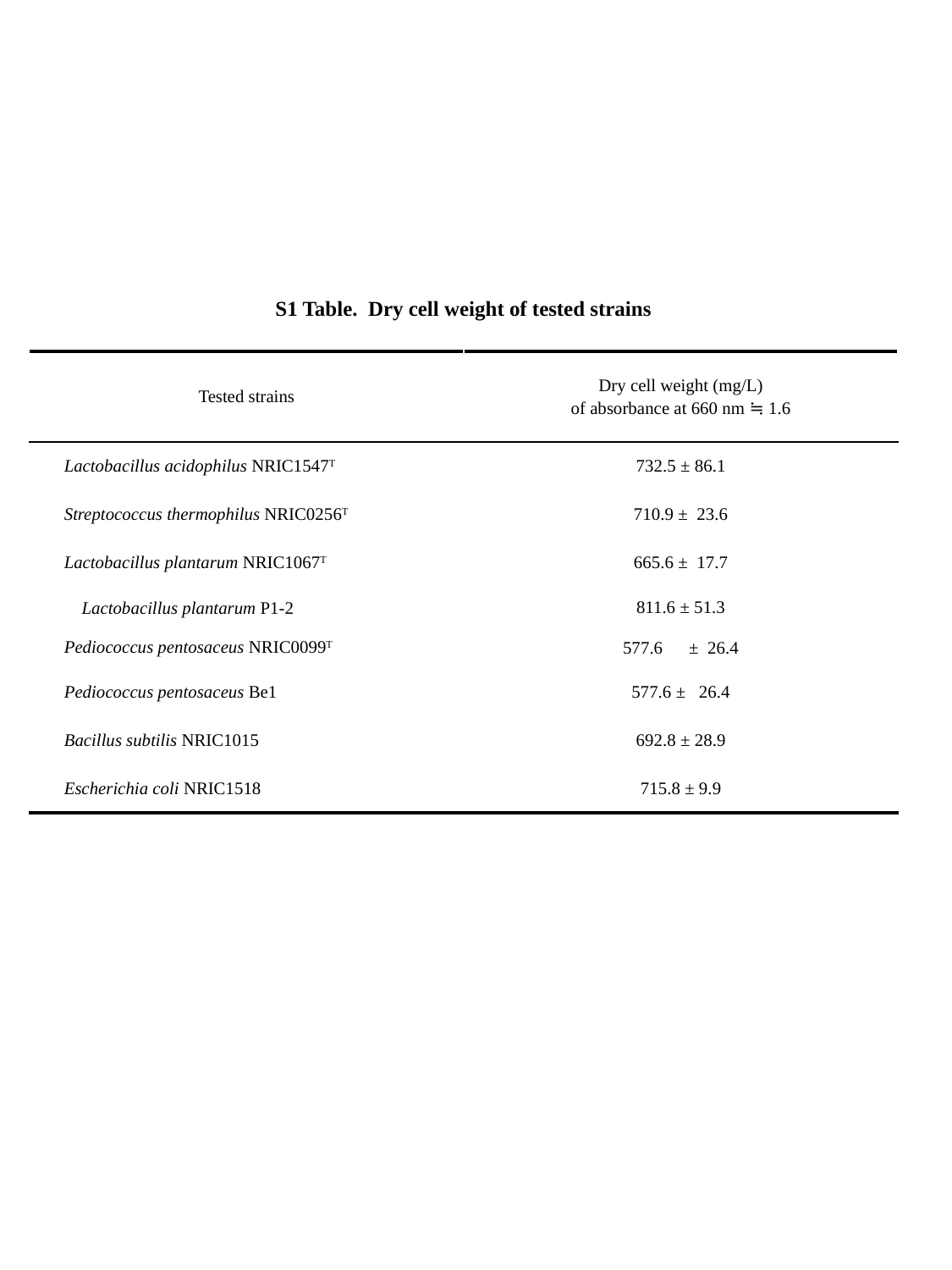

S1 Table. Dry cell weight of tested strains
| Tested strains | Dry cell weight (mg/L) of absorbance at 660 nm ≒ 1.6 |
| --- | --- |
| Lactobacillus acidophilus NRIC1547T | 732.5 ± 86.1 |
| Streptococcus thermophilus NRIC0256T | 710.9 ± 23.6 |
| Lactobacillus plantarum NRIC1067T | 665.6 ± 17.7 |
| Lactobacillus plantarum P1-2 | 811.6 ± 51.3 |
| Pediococcus pentosaceus NRIC0099T | 577.6　± 26.4 |
| Pediococcus pentosaceus Be1 | 577.6 ± 26.4 |
| Bacillus subtilis NRIC1015 | 692.8 ± 28.9 |
| Escherichia coli NRIC1518 | 715.8 ± 9.9 |
