# Supplementary material for: Isolation of lactic acid bacteria capable of reducing environmental alkyl and fatty acid hydroperoxides, and the effect of their oral administration on oxidative-stressed nematodes and rats
Source: PLoS One. 2020 Feb 27;15(2):e0215113. doi: 10.1371/journal.pone.0215113 (PMC7046221; doi:10.1371/journal.pone.0215113)
Supplement: S2 Table — (PPTX) [file pone.0215113.s009.pptx]

## Slide 1
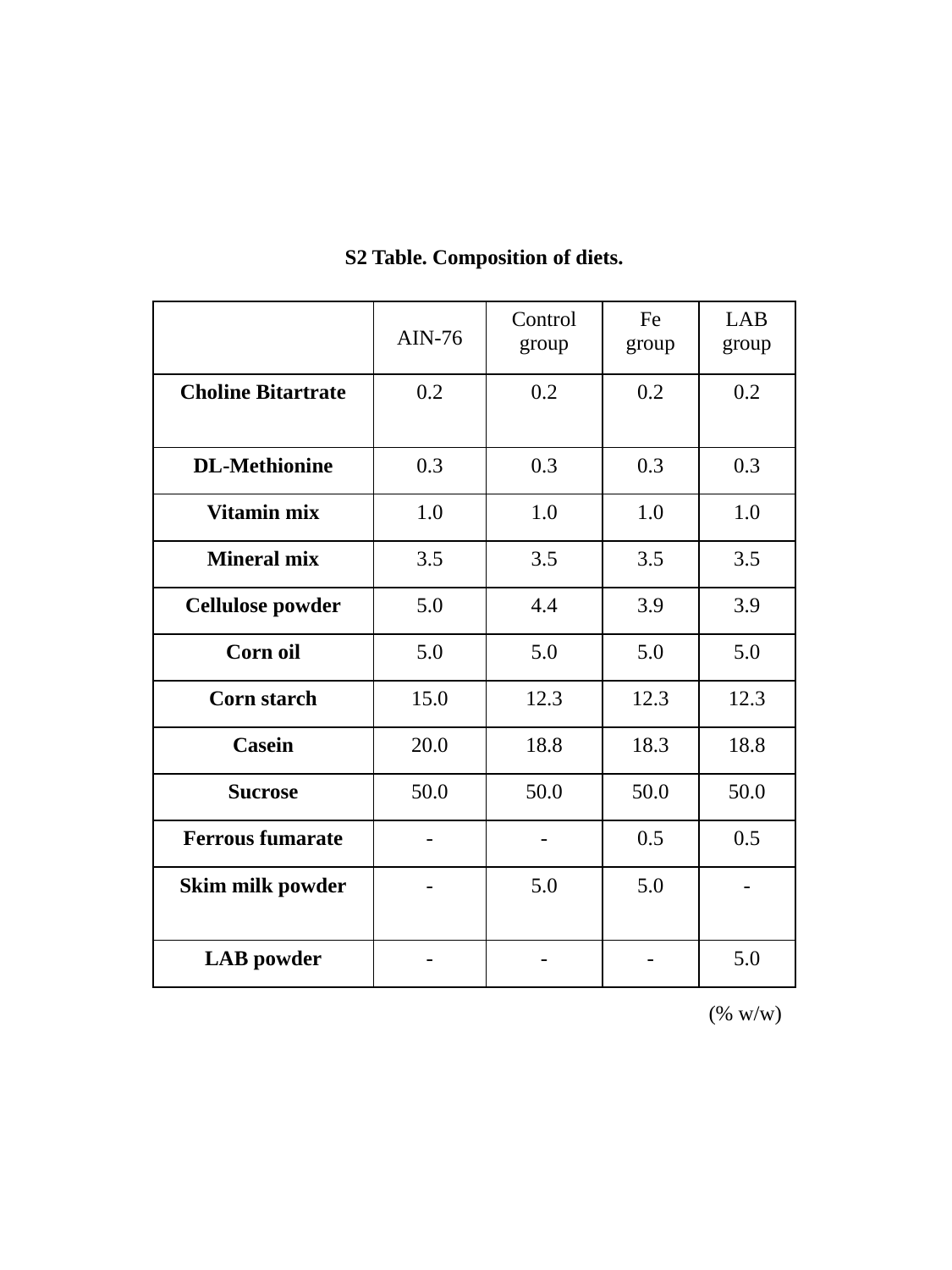

S2 Table. Composition of diets.
| | AIN-76 | Control group | Fe group | LAB group |
| --- | --- | --- | --- | --- |
| Choline Bitartrate | 0.2 | 0.2 | 0.2 | 0.2 |
| DL-Methionine | 0.3 | 0.3 | 0.3 | 0.3 |
| Vitamin mix | 1.0 | 1.0 | 1.0 | 1.0 |
| Mineral mix | 3.5 | 3.5 | 3.5 | 3.5 |
| Cellulose powder | 5.0 | 4.4 | 3.9 | 3.9 |
| Corn oil | 5.0 | 5.0 | 5.0 | 5.0 |
| Corn starch | 15.0 | 12.3 | 12.3 | 12.3 |
| Casein | 20.0 | 18.8 | 18.3 | 18.8 |
| Sucrose | 50.0 | 50.0 | 50.0 | 50.0 |
| Ferrous fumarate | - | - | 0.5 | 0.5 |
| Skim milk powder | - | 5.0 | 5.0 | - |
| LAB powder | - | - | - | 5.0 |
(% w/w)
