# Supplementary material for: Isolation of lactic acid bacteria capable of reducing environmental alkyl and fatty acid hydroperoxides, and the effect of their oral administration on oxidative-stressed nematodes and rats
Source: PLoS One. 2020 Feb 27;15(2):e0215113. doi: 10.1371/journal.pone.0215113 (PMC7046221; doi:10.1371/journal.pone.0215113)
Supplement: S3 Table — (PPTX) [file pone.0215113.s010.pptx]

## Slide 1
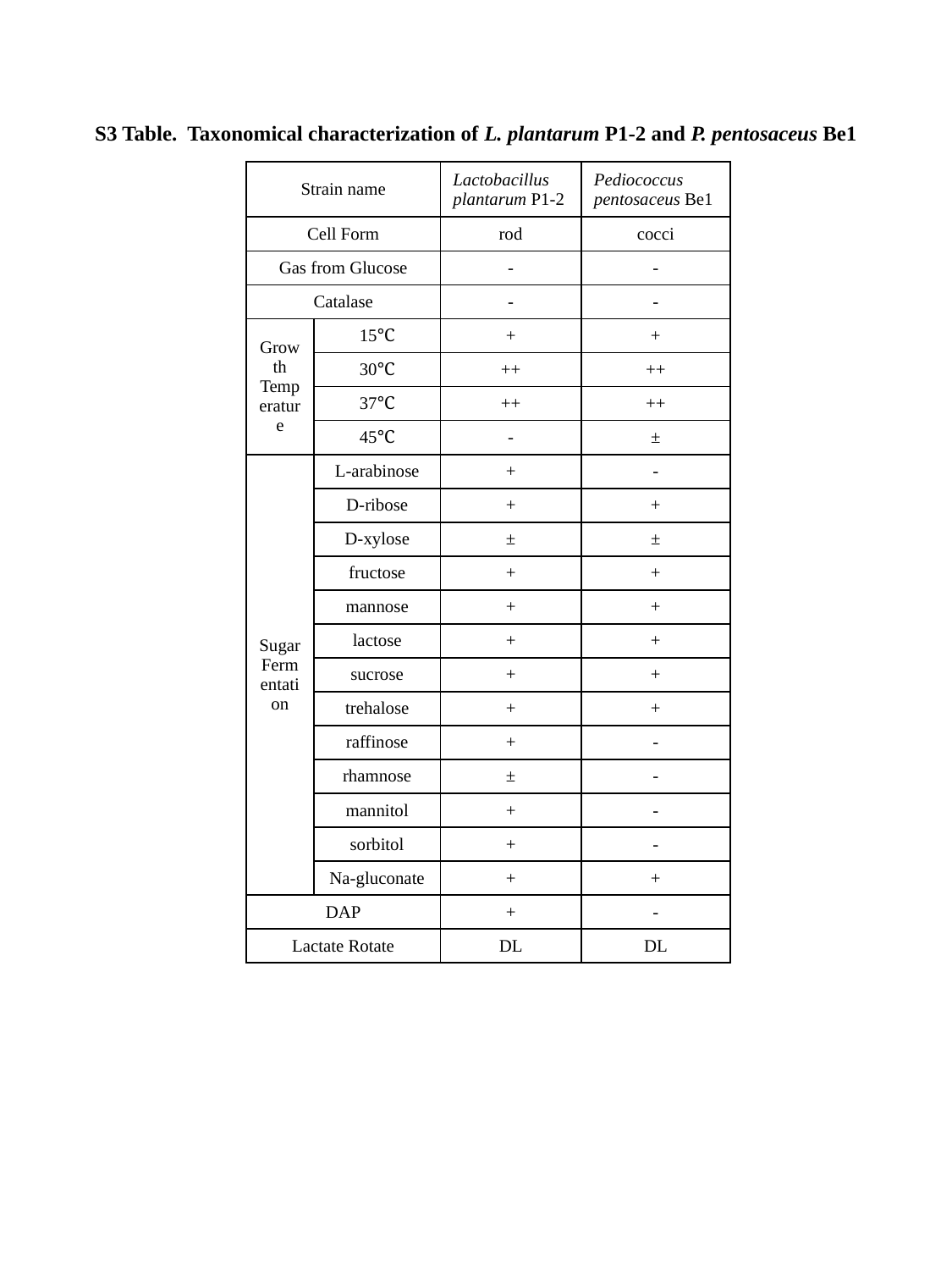

S3 Table. Taxonomical characterization of L. plantarum P1-2 and P. pentosaceus Be1
| Strain name | | Lactobacillus plantarum P1-2 | Pediococcus pentosaceus Be1 |
| --- | --- | --- | --- |
| Cell Form | | rod | cocci |
| Gas from Glucose | | - | - |
| Catalase | | - | - |
| Growth Temperature | 15℃ | + | + |
| | 30℃ | ++ | ++ |
| | 37℃ | ++ | ++ |
| | 45℃ | - | ± |
| Sugar Fermentation | L-arabinose | + | - |
| | D-ribose | + | + |
| | D-xylose | ± | ± |
| | fructose | + | + |
| | mannose | + | + |
| | lactose | + | + |
| | sucrose | + | + |
| | trehalose | + | + |
| | raffinose | + | - |
| | rhamnose | ± | - |
| | mannitol | + | - |
| | sorbitol | + | - |
| | Na-gluconate | + | + |
| DAP | | + | - |
| Lactate Rotate | | DL | DL |
